# Flexible Gas Sensor Based on PANI/WO_3_/CuO for Room-Temperature Detection of H_2_S

**DOI:** 10.3390/s25092640

**Published:** 2025-04-22

**Authors:** Dongxiang Zhang, Yingmin Liu, Yang Wang, Zhi Li, Dongkun Xiao, Tianhong Zhou, Mojie Sun

**Affiliations:** 1 School of Chemical Engineering, Northeast Electric Power University, Jilin 132012, China; zhangdongxiang1999@163.com (D.Z.); 15643553580@163.com (Y.L.); lz15304317120@163.com (Z.L.); xiao1999925@163.com (D.X.); zth159481@163.com (T.Z.); 2 State Key Laboratory of Precision Measuring Technology and Instruments, Tianjin University, Tianjin 300072, China; wangyang230410@163.com

**Keywords:** flexible gas sensor, PANI, H_2_S, ternary composite

## Abstract

Polyaniline (PANI) is currently one of the most extensively studied conductive polymers in the field of flexible gas sensors. However, sensors based on pure PANI generally suffer from problems such as low sensitivity and poor stability. To address these issues, in this work, a room-temperature hydrogen sulfide gas sensor of polyaniline/tungsten oxide/copper oxide (PANI/WO_3_/CuO) was synthesized using in situ polymerization technology. This gas sensor displays a response value of 31.3% to 1 ppm hydrogen sulfide at room temperature, with a response/recovery time of 353/4958 s and a detection limit of 100 ppb. Such an excellent performance is attributed to the high surface area and large adsorption capacity of the ternary composite, as well as the multi-phase interface synergistic effect.

## 1. Introduction

As industrialization keeps advancing, people are increasingly exposed to a more intricate variety of gases. Among them, toxic, harmful, flammable, and explosive gases have drawn special attention. This is because they not only have negative effects on the ecological environment but also pose risks to human life safety. Hydrogen sulfide (H_2_S), a colorless, acidic, and toxic gas with the distinct rotten-egg smell, is widespread in chemical production processes like coal combustion [1], oil refining [2], and metal smelting [3]. It also exists in natural environments, such as biogas, natural gas, wastewater, and volcanic gases [4,5]. In the power industry, H_2_S is one of the key decomposition products of the insulation medium sulfur hexafluoride (SF_6_) [6]. When the air humidity is high, H_2_S can corrode the metal parts of industrial equipment, causing cracks in structural materials and, thus, shortening the equipment’s lifespan. In daily life, incidents of H_2_S causing harm are quite common. For example, in 2021, an H_2_S leakage incident happened at Jilin Chemical Fiber Company, resulting in the accidental-inhalation-related deaths of five people. In 2022, two maintenance workers in Denmark died of poisoning from a high concentration of hydrogen sulfide gas caused by the decomposition of the vegetable oil remaining in the cargo compartment [7]. Syngas generated by coal gasification technology is the core fuel source of the power generation industry. This process is accompanied by the formation of a large number of sulfur-containing by-products, among which hydrogen sulfide accounts for the highest proportion [8]. When the concentration is extremely high, the human body may show symptoms like headache, nausea, and olfactory disorder, and even have its life safety endangered [9]. Therefore, it is of great significance that we achieve the continuous and accurate detection of H_2_S. In recent years, the gas-sensing method has become a popular area of exploration in the H_2_S detection field due to its advantages such as simple gas sensor fabrication, small size, low cost, and easy integration. The gas sensor can accurately detect a specific gas and its concentration. Its detection principle is to trigger a chemical characteristic response from the gas and convert it into a detectable electrical signal [10].

Gas sensors are categorized by operating mechanism into semiconductor-based [11], electrochemical [12], optical [13], and field-effect types [14]. Electrochemical sensors typically suffer from prolonged response/recovery times and poor stability, while optical sensors are limited by bulky size, high cost, and lengthy measurement cycles. Field-effect sensors, although promising, face challenges in complex fabrication processes and stringent signal amplification requirements. In contrast, metal-oxide semiconductor (MOX) sensors stand out for their high sensitivity, rapid response, and compact form factor. However, their reliance on high operating temperatures and rigid architectures restricts deployment in wearable, intelligent applications that demand flexibility and adaptability.

To address these limitations, researchers have focused on developing novel flexible wearable gas detection devices. For example, Zhang et al. [15] deposited reduced graphene oxide/tin dioxide (RGO/SnO_2_)-nanocomposite-sensing materials onto polyimide (PI) substrates, constructing a room-temperature flexible NO_2_-monitoring system. This compact system can be seamlessly integrated into face masks and smartwatches, demonstrating the practical implementation of flexible gas sensors in wearable electronics. Gao et al. [16] engineered zinc-oxide-modified single-walled carbon nanotubes (SWCNTs) as sensing materials on flexible nylon fibers, which were further integrated into face masks to enable the selective detection of ammonia (NH_3_) and other target gases. Li et al. [17] created stretchable, twistable, and bendable conductive sensing fibers by embedding reduced graphene oxide/zinc oxide (RGO/ZnO) nanosheets into cotton/elastic threads. These fibers exhibit excellent wearability and knittability, alongside outstanding NO_2_-sensing performance at ambient temperatures, showcasing promising potential for scalable textile-integrated gas-sensing solutions.

Gas-sensitive materials are the most crucial part of a gas sensor, and their research progress has a profound impact on the development trend of gas sensors. In the continuous wave of scientific and technological progress, the development of gas-sensitive materials shows remarkable stage-based features, evolving from the initial small-molecule inorganic materials to polymer-conductive polymers, from single-monomer materials to multi-component composite materials, and from simple doping forms to nanocomposite structures [18]. In 1987, MacDiarmid et al. [19] first reported the protonation/deprotonation properties of PANI, sparking early interest in exploring its potential as a sensitive material for gas sensor applications. However, pure PANI-based sensors exhibit critical limitations, such as a low response to H_2_S and poor long-term stability. Current strategies to address these challenges focus on optimizing PANI synthesis processes to leverage its intrinsic advantages while incorporating nanomaterial doping to create composite systems with synergistic effects. This approach aims to enhance gas molecule adsorption through complementary interactions between PANI and other functional materials [20].

Commonly used nanomaterials in such composites include metals, semiconducting metal oxides, and carbon-based materials [21,22,23,24,25,26,27]. Recent years have seen substantial advancements in flexible gas sensors fabricated from these PANI-based composites, driven by their adaptability for wearable and portable devices. Table 1 summarizes the latest international research progress on PANI-derived gas-sensitive materials for H_2_S detection, highlighting key performance metrics that address the limitations of pure PANI through material hybridization and structural innovation. Compared with other PANI-based H_2_S sensors, this work presents distinct advantages: room-temperature operation, reproducible response behavior, high flexibility enabled by the PET substrate, and excellent H_2_S response performance derived from the PANI/WO_3_/CuO ternary composite. The synergistic effects within the ternary heterostructure enhance gas adsorption kinetics and electrical conductivity modulation, addressing the common limitations of conventional PANI-based sensors (e.g., high operating temperature, and poor mechanical adaptability) while achieving superior sensitivity and stability under ambient conditions.

## 2. Materials and Methods

### 2.1. Synthesis of WO_3_ Microspheres

Four millimoles of Tungsten Hexachloride (WCl_6_) were added to 30 mL of glacial acetic acid. The resultant mixture was stirred continuously for 30 min. Subsequently, the solution was transferred into a 50-mL high-pressure reaction vessel. The reaction was then carried out at a temperature of 100 °C for a period of 12 h. After the reaction, the product was allowed to cool to room temperature. It was then washed three times successively with deionized water and absolute ethanol. Following the washing process, the product was dried under vacuum conditions at 60 °C for 12 h. Once the drying was completed, the product was removed and placed in a muffle furnace. The temperature within the furnace was increased at a rate of 2 °C per minute until it reached 450 °C. The product was calcined at this temperature for 2 h. Finally, it was cooled down to room temperature, thereby obtaining the yellow WO_3_ material.

### 2.2. Synthesis of WO_3_/CuO Nanocomposite

The synthesis procedure was executed as follows: 200 mg of WO_3_ microsphere powder was dissolved in a 30-mL solution consisting of absolute ethanol and deionized water at a volume ratio of 1:1. The solution was magnetically stirred for 30 min. Subsequently, 0.0348 g of Cu(NO_3_)_2_·3H_2_O was added, and the mixture was stirred for an additional 30 min. The molar ratio of copper to tungsten is 1:6. The solution was then transferred into a 50-mL stainless-steel autoclave internally lined with tetrafluoroethylene. The reaction was conducted at 180 °C for 5 h. After the reaction reached completion, the solution was permitted to cool spontaneously to room temperature. The solution was then subjected to centrifugation and washed three times with deionized water and absolute ethanol in sequence. Finally, the product was dried at 70 °C for 8 h to yield the final product material.

### 2.3. Synthesis of PANI/WO_3_/CuO Nanocomposites and Preparation of Sensitive Elements

**Figure 1:**
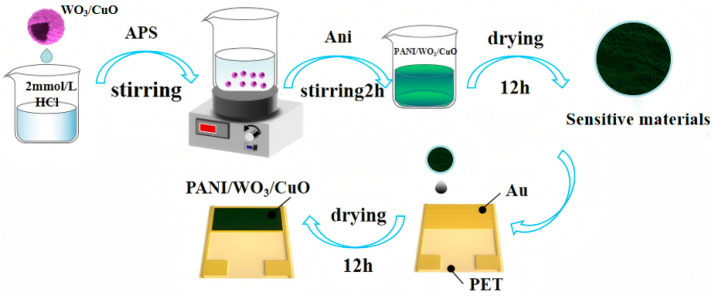
Material synthesis and sensitive element preparation.

As depicted in the Figure 1, 1 mmol of ammonium persulfate (APS) was introduced into 10 mL of 2 mol/L hydrochloric acid (HCl) solution. The mixture was continuously stirred for a duration of 30 min. Subsequently, the solution was pre-cooled in an ice-water bath to yield Solution A. The WO_3_/CuO nanomaterials were added to 10 mL of 2 mol/L HCl. The resulting suspension was subjected to sonication for 10 min to ensure homogeneous dispersion. Thereafter, 93 μL (equivalent to 0.1 mmol) of aniline monomer was added. The mixed solution was further sonicated for 30 min and then pre-cooled to obtain Solution B. The molar ratios of WO_3_/CuO to aniline monomer were set as n(WO_3_/CuO):n(Ani) = 0%, 5%, 10%, 20%, and 50%, which were, respectively, denoted as PWC0, PWC5, PWC10, PWC20, and PWC50. Subsequently, Solution A was slowly poured into Solution B, and the reaction was conducted in an ice-water bath for 2 h. The resultant product was washed three times with deionized water and absolute ethanol, respectively. Thereafter, it was vacuum-dried at 60 °C for 12 h. The dried material was thoroughly ground, and an appropriate quantity of terpineol was added. The material was then spin-coated onto a PET gold interdigitated electrode and dried at room temperature for 24 h to fabricate the flexible gas sensor.

A proper amount of the prepared sample is placed in an agate mortar. Add 2–3 drops of terpineol and the sample is ground until it turns into a slurry. Then, the paste was evenly coated on the surface of the fork electrode with a fine brush of 0.3 mm diameter. Then, the sample is subjected to drying in a vacuum drying oven maintained at a temperature of 70 °C for a duration of 12 h; the sensitive element of the flexible gas sensor is obtained. In this paper, polyethylene terephthalate (PET) is employed as the substrate material of the sensor. Subsequently, through the methods of photolithography and magnetron sputtering, a layer of Au electrode (100 nm) is deposited on the PET. The IDE structure adopted in this paper has an electrode size of 10 mm × 10 mm. The line width and the spacing of the interdigitated electrodes are both 100 μm, and the number of interdigitated electrode pairs is 10. These interdigitated electrodes are provided by Guangzhou Yuxin Sensing Co., Ltd. in Guangzhou, China.

## 3. Results

### 3.1. Materials Characterizations

The identity of the prepared nanocomposite as PANI/WO_3_/CuO was affirmed through comprehensive measurements involving X-ray diffraction (XRD), scanning electron microscopy (SEM), transmission electron microscopy (TEM), energy-dispersive X-ray spectroscopy (EDS), and X-ray photoelectron spectroscopy (XPS). For the XRD analysis, an XRD-7000 X-ray diffractometer, manufactured by Shimadzu Corporation, Kyoto, Japan, was utilized. The diffraction experiment was carried out with the Kα radiation of a Cu target, having a wavelength (λ) of 1.5406 Å. The operating conditions included a tube voltage of 40 kV and a current of 20 mA, and the angular range of 2θ was scanned from 10° to 80°. The scanning electron microscopy investigation was conducted using the SU8220 model supplied by Hitachi, Ltd., Kyoto, Japan, with an operating voltage set at 20 kV. The field-emission transmission electron microscopy analysis was performed with the FEI TECNAI G2 F20 instrument from FEI Company, Hillsboro, OR, USA. Equipped with multiple accessories such as energy-dispersive X-ray spectroscopy (EDX) and high-angle annular dark-field (HAADF) detectors, dark-field images, high-resolution images, and selected area electron diffraction patterns were acquired for an in-depth EDX energy spectrum analysis. The X-ray photoelectron spectroscopy measurements were carried out using the AXIS Supra model from Kratos Analytical Ltd., Manchester, UK, with a monochromatized Al Kα radiation source (hυ = 1486.6 eV).

**Figure 2:**
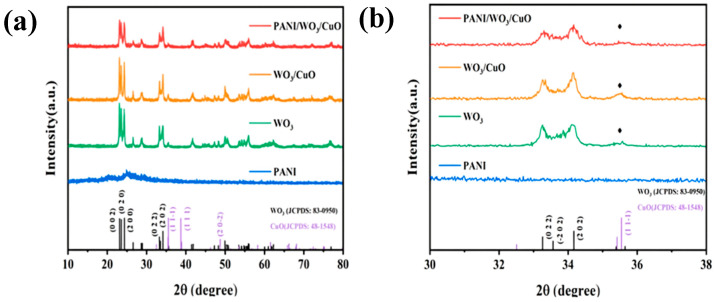
(**a**) XRD patterns of PANI, WO_3_, WO_3_/CuO, and PWC10 (2θ = 10–80°); and (**b**) XRD pattern of PANI/WO_3_/CuO (2θ = 30–38°).

As shown in Figure 2a, PANI exhibits a characteristic X-ray diffraction peak at 2θ = 25.105°, which arises from the periodic alignment of its molecular chains. The XRD patterns of pure WO_3_ and the WO_3_/CuO composite are analyzed to characterize their crystal structures and phase purities. For the as-synthesized WO_3_, distinct diffraction peaks at 23.105°, 23.580°, 24.346°, 33.258°, and 34.151° correspond to the (002), (020), (200), (022), and (202) planes of monoclinic tungsten trioxide, respectively. These peaks match well with the standard JCPDS card (No. 83-0950) [28], confirming the high-phase purity of the WO_3_ sample. In the case of the WO_3_/CuO composite, a prominent diffraction peak emerges at 2θ = 35.505°, with an increased intensity compared to the pure WO_3_ spectrum. As illustrated in Figure 2b, this peak is indexed to the (11-1) plane of CuO (JCPDS No. 48-1548) [29], indicating the successful formation of the heterostructured WO_3_/CuO composite. The presence of this CuO-specific peak verifies the integration of copper oxide into the tungsten trioxide matrix, highlighting the successful synthesis of the binary composite material.

**Figure 3:**
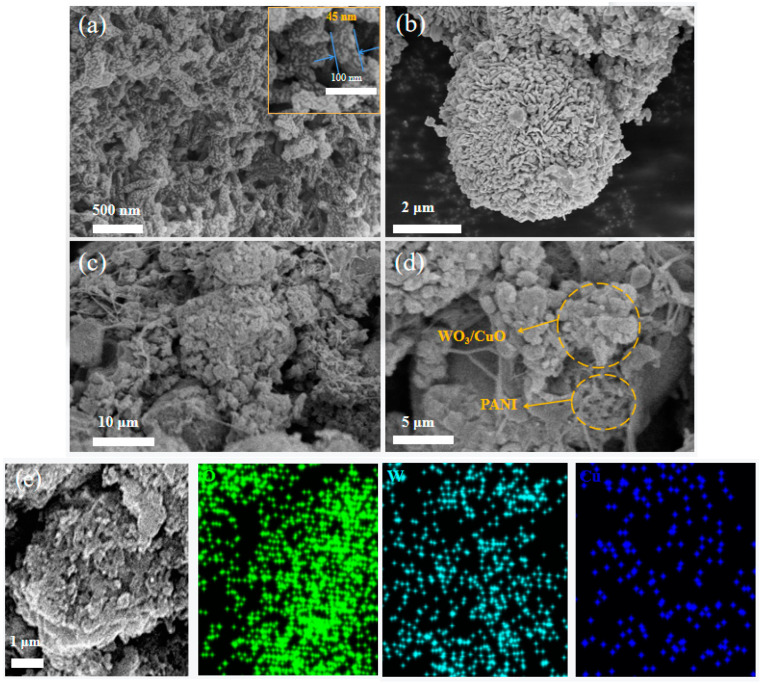
(**a**) PANI nanofibers; (**b**) spherical WO_3_/CuO; (**c**,**d**) SEM images of PWC10; and (**e**) EDS elemental distribution maps of W, O, and Cu in the PWC10 composite.

The morphological and microstructural characteristics of the synthesized PANI, spherical WO_3_/CuO, and PWC10 composites were investigated using a scanning electron microscope (SEM). As depicted in Figure 3a, the SEM image of PANI reveals a uniform and continuous-network-structured nanofiber morphology. The SEM image of the spherical WO_3_/CuO (Figure 3b) indicates that the fabricated spherical WO_3_/CuO exhibits excellent dispersibility. Using Nano Measurer software(Version 1.2.5), five scanning electron microscopy (SEM) images were analyzed, with 50 valid particles selected from each image. The results, calculated as the mean ± standard deviation, determined the diameter of the PANI nanofibers to be (45 ± 8 nm) and the size of the WO_3_/CuO microspheres to be (4.7 ± 0.6 μm). Figure 3c,d display the SEM images of the PWC10 sample following the polymerization process. Evidently, the PANI nanofibers are interspersed among the spherical WO_3_/CuO composites, functioning as “bridges” to connect the WO_3_/CuO nanospheres.

Furthermore, an energy-dispersive X-ray spectroscopy (EDS) elemental analysis was performed on the PWC10 composite. The resulting elemental mapping of PWC10 is presented in Figure 3e. The detected elements encompass C, N, Cu, O, and W. The examined region, as illustrated in Figure 3e, assumes a spherical shape. From the figure, the distribution of the primary elements W, Cu, and O on the material surface can be distinctly observed, which provides conclusive evidence that WO_3_/CuO nanoparticles are grown on the surface of PANI.

**Figure 4:**
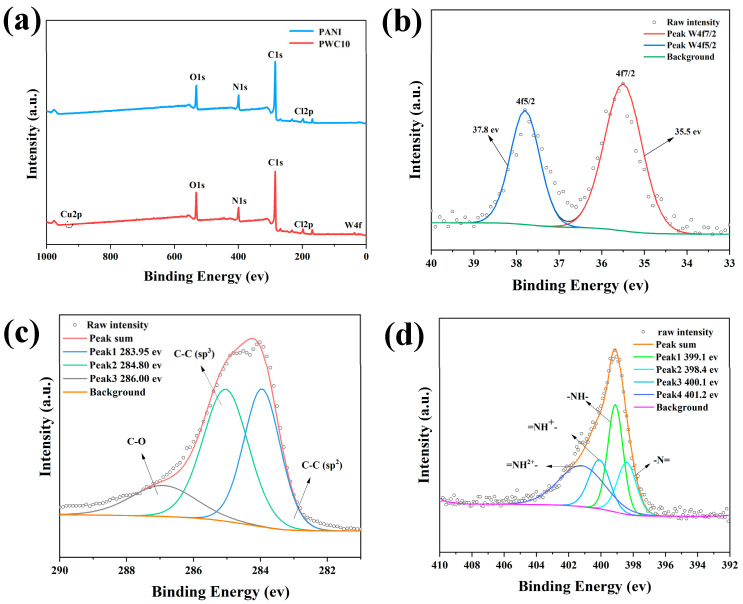
(**a**) The full XPS spectra of PANI and PWC10; and (**b**–**d**) high-resolution XPS spectra of W 4f, C 1s, and N 1s of PWC10.

In the in-depth exploration of PANI and polyaniline/tungsten trioxide/copper oxide (PANI/WO_3_/CuO) nanomaterials, X-ray photoelectron spectroscopy technology has played a crucial role. From the full spectra of the two materials shown in Figure 4a, it can be seen that the C 1s, N 1s, and O 1s peaks of PANI are located at 284.6 eV, 399.6 eV, and 531.6 eV, respectively. WO_3_ has characteristic W 4f peaks. In Figure 4b, its binding energy peaks appear at 35.5 eV and 37.8 eV, corresponding to the characteristic spin-orbit split states of W 4f7/2 and W 4f5/2, respectively [30].

Looking further at Figure 4c, the high-resolution C 1s spectrum of PANI/WO_3_/CuO is decomposed into three Gaussian-fitted peaks. The peak at 283.95 eV corresponds to the C-C(sp^2^) bond in the PANI backbone, and the peaks at 284.8 eV and 286.0 eV represent the C-C(sp^3^) bond and C-Cl bond, respectively. These conclusions are supported by references [31,32]. Then, looking at Figure 4d, the high-resolution N spectrum of PWC10 can be subdivided into four Gaussian-fitted peaks. Among them, quinoid amine (-N =) and benzenoid amine (-NH-) correspond to the peak positions of 398.4 eV and 399.1 eV in the composite. The peaks at 400.1 eV and 401.2 eV are attributed to the positively charged imine (=NH^+^-) in the bipolaron state and the protonated amine (-NH_2_^+^-) in the polaron state [33].

### 3.2. H_2_S-Sensing Performance

**Figure 5:**
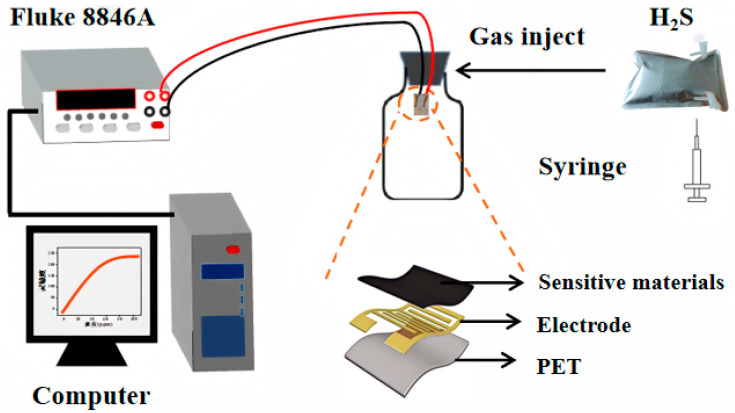
Schematic diagram of the test platform.

As shown in Figure 5, the experimental platform for the sensor performance test is mainly composed of gas sensors, gas to be measured, airbags, gas cylinders, syringes, computers, multimeter, wires, etc. We used a conductive silver paste to connect the two ends of the finger electrode with two copper wires, and clamped the other end of the copper wire with an electrode to form a test circuit. The PANI/WO_3_/CuO sensor was exposed to various concentrations of hydrogen sulfide at room temperature in a measured range of 100 ppb to 10 ppm. The real-time resistance of the sensor response was recorded by the Fluke 8846A data recorder.

The gas-sensing element consists of a flexible substrate (PET), gold interdigital electrodes, and a sensitive layer. A small amount of the synthesized material is added with water to form a slurry, and then the slurry is evenly coated on the surface of the interdigital electrodes. It is air-dried and left to stand at room temperature for 24 h to enhance its stability. The gas-sensing characteristics of this sensor were examined in a static test system. The tests were carried out under laboratory conditions (relative humidity of 20–40% and room temperature of 25 ± 5 °C).

During the test, a certain amount of test gas was injected into the closed system: 1 mL of gas was quickly injected into a glass bottle with a volume of 1 L from the air bag (concentration of 10 ppm) by syringe. The sensor was placed in the middle of the glass bottle, so that the sensor was exposed to the environment with a concentration of 10 ppm. The change in the sensor resistance is measured with a digital multimeter and transmitted to a computer. The stable resistance obtained in the gas being tested is denoted as Rg. The response value of the gas sensor in this paper is defined as the ratio of the resistance in air to the resistance in the presence of the target gas: Response (%) = (Ra − Rg)/Ra × 100%, where Ra represents the stable resistance value of the sensor in air and Rg represents the stable resistance value of the sensor when exposed to H_2_S. The time required for the component to reach 90% of the steady-state resistance after the adsorption and desorption of the target gas is defined as the response time and recovery time [34,35].

#### 3.2.1. Response/Recovery Characteristics

To compare and analyze the influence of the additional amount of WO_3_/CuO nanomaterials on the gas-sensing performance of the composite materials, the H_2_S gas-sensing characteristics at room temperature of flexible sensor devices using PANI- and PANI/WO_3_/CuO-composite-sensitive materials with different addition amounts of WO_3_/CuO were tested. Before the introduction of gas, the gas sensor must obtain a stable resistance Ra in the air. The initial baseline resistance in the air is stable at 105 ± 5 Ω, and the resistance gradually recovers to more than 90% of the baseline after gas removal.

**Figure 6:**
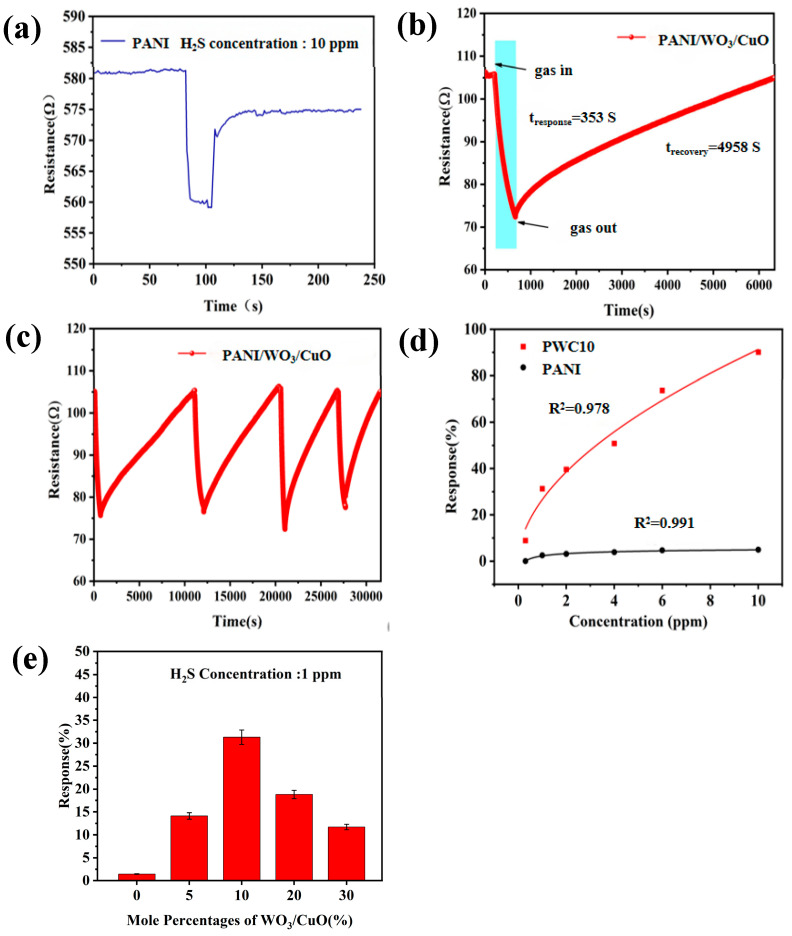
(**a**) Response–recovery curve of pure PANI to 10 ppm H_2_S gas at room temperature; (**b**) recovery curve of PANI/WO_3_/CuO to 1 ppm H_2_S gas at room temperature; (**c**) Continuous cycle response recovery curve of PANI/WO3/CuO to 1 ppm hydrogen sulfide at room temperature; (**d**) response curve of PANI/WO_3_/CuO to different concentrations of H_2_S at room temperature; and (**e**) effect of WO_3_/CuO addition (mole percentage) on the gas-sensitivity performance of PWC sensor.

Figure 6e shows the response characteristics of different devices to 1 ppm H_2_S at room temperature. The results demonstrate that the response value of the sensor based on the PANI-sensitive material to 1 ppm H_2_S at room temperature is 1.77%. The response value of the sensor shows a trend of “increase–peak–decrease” with the increase in the addition amount of WO_3_/CuO. The sensor based on the PWC10-sensitive material has the best gas-sensing performance, and its response value to 1 ppm H_2_S at room temperature can reach 31.3%. Figure 6a depicts the response–recovery curve of the pure PANI sensor. Its response value to 10 ppm H_2_S at room temperature is 4.13%. After the resistance value of this device decreases upon contact with H_2_S, it cannot fully recover to its initial state when placed in air. The gas-sensitive characteristics of the PWC10 sensor to H_2_S are much better than those of the pure PANI sensor. Its response value to 10 ppm H_2_S at room temperature can reach 90.1% (Figure 6e), which is more than twenty times that of the pure PANI sensor (4.13%) to 10 ppm H_2_S at room temperature, and it has a lower detection limit (100 ppb). The sensor has a response value of 8.7% at the limit detection of 100 ppb H_2_S, a response–recovery time of 64 s, and a recovery time of 738s. The detection limit (LOD) is calculated according to the international standard 3σ method: LOD = 3σ/S, where σ is the standard deviation of the blank signal (pure air), and S is the sensor’s sensitivity to a 100 ppb H_2_S response (ΔR/Ra). In the experiment, the σ of the blank signal was 0.12%, and the measured sensitivity at 100 ppb was S = 8.7%. Therefore, LOD = 3 × 0.12%/8.7% ≈ 41 ppb. To ensure the reproducibility of the experiment, it is conservatively reported as 100 ppb. Figure 6c shows the continuous cyclic response–recovery characteristic curve of the sensor based on the PWC10-sensitive material to 1 ppm H_2_S at room temperature. This indicates that the sensor based on the PWC10-sensitive material can be reused for the continuous detection of H_2_S. Moreover, the “response value–gas concentration” fitting curves of the PANI sensor and the PWC10 sensor shown in Figure 6d conform to the typical exponential model [36]. The calibration curve in Figure 6d was fitted by the isothermal line of Freundlich (power law function: y = a × x^ (−b)), where the R^2^ of PWC10 was 0.992 and that of PANI was 0.914.

#### 3.2.2. Selectivity and Stability

**Figure 7:**
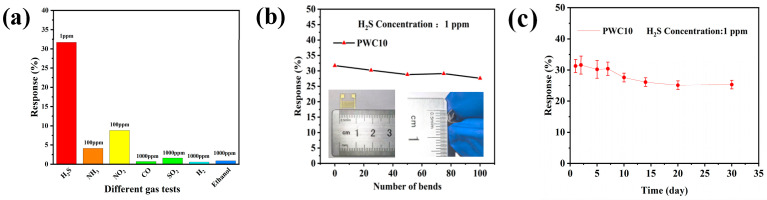
(**a**) Response values of PANI/WO_3_/CuO to various gases at room temperature; (**b**) response of the PWC10 sensor under different bending cycles; and (**c**) variation in response of the PWC10 sensor over 30 days.

Selectivity, a key metric in gas-sensing features, was probed for the sensor employing the PWC10-sensitive material, which exhibits the highest response magnitude. Figure 7a illustrates the responses of this sensor to diverse gases at ambient temperature, such as NH_3_, ethanol, hydrogen, carbon monoxide, sulfur dioxide, and NO_2_. To assess the device’s stability, both bending endurance and longevity tests were carried out. As presented in Figure 7b, when the PWC10 sensor is subjected to different bending cycles (0, 25, 50, 75, and 100 times), its response to 1 ppm H_2_S experiences minimal variation. This outcome implies that the PWC10 ternary sensor possesses excellent bending resilience. Moreover, the lifespan represents an essential factor in evaluating sensor performance. In this study, the response decay of the PWC10 sensor to 1 ppm H_2_S over a span of 30 days was explored. Figure 7c reveals that the sensor’s response value displays a declining tendency during the test on the third day, and then stabilizes in subsequent trials. The primary factors contributing to the reduction in the sensor’s response value are device deterioration and the elimination of certain unstable adsorption sites. The results show that the performance of the PWC10 ternary H_2_S sensor does not deteriorate substantially over time, signifying good stability.

#### 3.2.3. Humidity Effect

**Figure 8:**
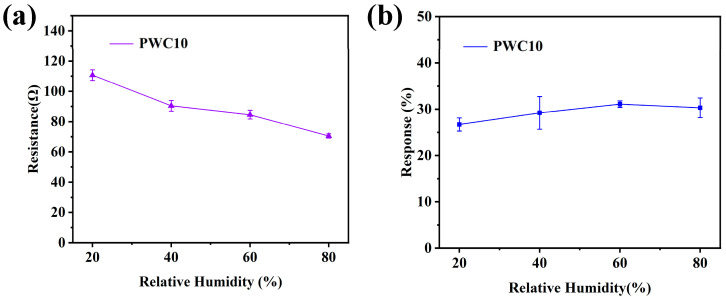
Sensor based on PWC10-sensitive material at room temperature for initial resistance in (**a**) air and (**b**) sensor response values in the humidity range of 1 ppm H_2_S 20–80%.

Humidity is a crucial factor influencing gas sensors. To explore its impact on the sensor, we utilized a humidifier, a dryer, and an electronic device capable of real-time humidity display to control the humidity environment. Figure 8a,b illustrate the initial resistance and the sensitivity to 1 ppm H_2_S of the sensor with PWC10-sensitive material within a relative humidity (RH) range of 20–80%.The experimental results reveal a notable trend. As the relative humidity rises, the initial resistance of the sensor decreases, and its response to H_2_S improves. However, when the humidity exceeds 60% and reaches 80%, the sensor’s response value starts to decline. The variation in the sensor’s response to H_2_S with respect to RH can be elucidated from the perspectives of surface adsorption behavior and mass transfer processes.

The decrease in the initial resistance can be attributed to the surface adsorption of water molecules and the enhancement in electrical conductivity. The surfaces of sensor materials, such as the metal-oxide semiconductor WO_3_/CuO heterojunction or the conductive polymer PANI, are rich in polar groups like hydroxyl and amine groups. As the humidity increases, water molecules adhere to the material surface through hydrogen bonding or physical adsorption, forming a thin water film. In the case of metal oxides (MOXs), the dissociation of water generates H^+^/OH^−^ ions, which enhances the surface ionic conductivity. For conductive polymers like PANI, water molecules act as a “plasticizer”, reducing the resistance between molecular chains and increasing the carrier mobility, thereby lowering the resistance [37].

An appropriate amount of water molecules creates active adsorption sites on the material surface, which interact synergistically with H_2_S molecules. Water molecules enhance the surface adsorption of H_2_S through hydrogen bonding or dipole–dipole interactions. For example, H_2_S dissolves in the water film to form HS^−^/S^2−^ ions, accelerating the redox reaction with the metal oxide. Additionally, the increased humidity makes the material surface more moist, optimizing the charge-transfer efficiency at the gas–solid interface and improving the sensor’s sensitivity to H_2_S.

When the RH exceeds 60% and reaches 80%, the response value drops. At high humidity levels, water molecules occupy the preferential adsorption sites on the sensor surface, such as the oxygen vacancies in metal oxides and the polar groups in PANI. This leads to a reduction in the effective adsorption of H_2_S. Moreover, the increased thickness of the water film creates a diffusion barrier. H_2_S gas has to pass through a thicker water layer to reach the active surface of the material, increasing the mass-transfer resistance and reducing the reaction efficiency [38].

### 3.3. H_2_S Gas-Sensing Mechanism

In terms of microstructure, the porous nanostructure of the PANI/WO_3_/CuO heterojunction significantly bolsters its H_2_S sensing performance. Scanning electron microscopy (SEM) and transmission electron microscopy (TEM) disclose that the in situ polymerized PANI/WO_3_/CuO nanocomposites are tightly interwoven, giving rise to a porous nanostructure with a large specific surface area. It is worth noting that the adsorption capacity of the PANI composite is 5–10 times higher than that of pure PANI [39]. The absorption process of hydrogen sulfide by the polyaniline–copper oxide–tungsten oxide ternary composite at room temperature also involves multi-phase interface synergistic effects, and its reaction mechanism can be elucidated from the following three aspects:

PANI + H_2_S → PANI-H^+^ + HS^−^ (1)

3WO_3_ + 7H_2_S → 3WS_2_ + SO_2_ + 7H_2_O + 3Vo + 6e^−^ (2)

Vo + H_2_S → Vo (H_2_S)ads (3)

H_2_S (ads) → 2H^+^ + S^2−^ (4)

CuO(s) + H_2_S(g) → CuS(s) + H_2_O(g) (5)

#### 3.3.1. Interface Activation Mechanism of Polyaniline

Polyaniline (PANI) features a typical π–π conjugate system, endowing it with distinct physical and chemical properties. The interaction of its internal π–electron clouds gives the material surface a strong affinity for H_2_S molecules, constantly attracting and aggregating H_2_S molecules on its surface. This excellent conductivity plays a crucial role throughout the reaction process, facilitating the migration of H^+^. When PANI comes into contact with H_2_S, the imine groups (-N =) in PANI molecules exhibit high chemical reactivity and engage in a protonation reaction with H_2_S. As shown in Equation (1), in this reaction, one hydrogen atom in the hydrogen sulfide molecule separates and binds to the imino group of PANI to form a positively charged PANI-H^+^ and produce HS^−^ ions. This protonation reaction has far-reaching implications, directly distorting the energy-band structure of polyaniline. The originally regular energy-band structure is disrupted, the electron distribution is significantly altered, and the Fermi level shifts towards the low-energy end. As the reaction progresses, a large number of PANI-H^+^ are generated and accumulate on the material surface, thus forming a hole-accumulation layer on the PANI surface. The emergence of this hole-accumulation layer modifies the electrical properties of the material surface, exerting a profound influence on subsequent charge transfer and reaction processes [40].

#### 3.3.2. Redox Synergy of CuO/WO_3_ Heterojunction

The surface of the CuO/WO_3_ heterojunction is abundant in numerous active sites, which lays the foundation for its interaction with H_2_S molecules. Due to the differences in the crystal structures of CuO and WO_3_ and the electron states of surface atoms, a unique electric field and electron–cloud distribution are formed at the heterojunction interface. This special micro-environment enables H_2_S molecules to be adsorbed onto the heterojunction surface through both physical and chemical adsorption. The adsorbed H_2_S molecules trigger a charge-transfer phenomenon. The conduction-band position of WO_3_ is relatively high, while the valence-band position of CuO is relatively low. Within the heterojunction system, electrons transfer from the conduction band of WO_3_ to the valence band of CuO, forming a built-in electric field. Once H_2_S molecules are adsorbed, due to their reducing nature, they react with the surface active oxygen species. The sulfur atoms in H_2_S capture the electrons of the surface oxygen atoms, reducing the surface oxygen atoms and oxidizing H_2_S itself. This process causes electrons to transfer from H_2_S to the heterojunction surface, thereby altering the charge distribution within the heterojunction and laying the foundation for subsequent reactions. In terms of electron–hole recombination, when H_2_S molecules are adsorbed on the heterojunction surface and react, electron transfer occurs. These electrons participate in the recombination with holes. Originally, there is a certain dynamic equilibrium of electron–hole pairs in the heterojunction. The extra electrons introduced by H_2_S break this equilibrium, increasing the probability of electron–hole recombination. As recombination occurs, the number of charge carriers participating in conduction within the material decreases, macroscopically manifested as an increase in resistance. This change in resistance is closely related to the sensing performance of the sensor. In the detection of H_2_S gas, the sensor perceives the change in gas concentration by detecting changes in electrical properties such as resistance. Many studies have reported this in semiconductor heterojunction gas sensors [41].

#### 3.3.3. Oxygen-Vacancy-Mediated Deep-Purification Mechanism

The oxygen vacancies (V_0_) on the surface of WO_3_ play a vital role in its interaction with H_2_S gas. Acting as Lewis acid sites, the oxygen vacancies, with their unsaturated electron structures, show a strong affinity for H_2_S molecules and can efficiently capture H_2_S molecules through coordination. As shown in Equations (2)–(4), when H_2_S molecules are adsorbed near the oxygen vacancies on the WO_3_ surface, the originally stable structure of H_2_S molecules is affected by the special electron-cloud environment around the oxygen vacancies, triggering a series of complex chemical reactions. In this process, the adsorbed H_2_S dissociates near the oxygen vacancies. Due to the change in the electron-cloud density at the oxygen vacancies, the chemical bonds in the H_2_S molecule are weakened, and the covalent bond between hydrogen and sulfur atoms breaks, releasing S^2−^ ions. Meanwhile, the Cu^2+^ ions in the system, due to their charge characteristics, rapidly combine with the released S^2−^ ions. As shown in Equation (5), this process is based on charge attraction and chemical affinity, leading to a chemical reaction that forms CuS precipitates. The formation of these precipitates not only changes the form of substances in the system but also further influences the electron distribution and chemical activity on the material surface. The H^+^ ions generated during the dissociation process embark on a migration journey through the unique proton-conduction channels of polyaniline. Polyaniline has a special conjugate structure, with proton-conduction paths existing in its molecular chain. Driven by the electric field and concentration difference, H^+^ ions gradually migrate to the material surface along the proton-conduction channels of polyaniline. On the material surface, H^+^ ions participate in subsequent reactions or maintain the charge balance of the system through interactions with other substances. As H_2_S molecules are continuously captured and dissociated, S^2−^ and Cu^2+^ continuously react to form CuS precipitates, and H^+^ ions continuously migrate, creating a cyclic process in the entire system. Considering the long-term stability of the sensor, oxygen vacancies play an important role. On one hand, an appropriate and stable number of oxygen vacancies can continuously and effectively adsorb H_2_S molecules, ensuring the sensor’s continuous detection ability for H_2_S gas. This is because, if the oxygen vacancies are unstable and prone to change, their adsorption capacity for H_2_S will be affected, thus influencing the sensor’s sensitivity. On the other hand, in the actual application environment, there are various interfering factors such as oxygen and humidity in the environment. Stable oxygen vacancies can reduce the impact of these interfering factors on the sensor’s performance. For example, when there is oxygen in the environment, unstable oxygen vacancies may react with oxygen, thus changing their adsorption characteristics for H_2_S. While stable oxygen vacancies can resist this interference to a certain extent, maintaining the relative stability of the H_2_S adsorption and dissociation process, thereby ensuring that the sensor can work stably for a long time [42,43].

In conclusion, the sensing mechanism of the PANI/WO_3_/CuO heterojunction sensor for H_2_S is a complex multi-process synergistic effect. The interface activation mechanism of polyaniline changes its own energy-band structure and surface electrical properties through protonation reactions; the redox synergy of the CuO/WO_3_ heterojunction realizes the sensing of H_2_S by influencing the resistance change through electron-hole recombination based on the special electric field and electron-cloud distribution at the interface; the oxygen-vacancy-mediated deep-purification mechanism utilizes the adsorption and dissociation of H_2_S by oxygen vacancies and subsequent ion reactions and migration processes to achieve the deep purification and sensing of H_2_S. These three mechanisms cooperate with each other to jointly improve the sensor’s sensing performance for H_2_S gas.

## 4. Conclusions

In conclusion, a new room-temperature flexible hydrogen sulfide sensor of the PANI/WO_3_/CuO ternary nanocomposite was prepared by in situ polymerization. The mesh nanofiber structure of gas-sensitive materials was confirmed by characterization The sensing characteristics of the PANI/WO_3_/CuO sensor for hydrogen sulfide gas were evaluated from the perspective of response–recovery, repeatability, stability, and bending resistance at room temperature. The PANI/WO_3_/CuO ternary composite achieves the efficient removal of H_2_S at room temperature through the synergistic mechanisms of hierarchical adsorption, heterojunction catalysis, and defect activation. This research furnishes a theoretical basis for the design of multi-component synergistic adsorbents.

The sensor’s room-temperature operation eliminates the need for external heating, making it ideal for flexible battery-powered systems such as smart protective clothing and intelligent masks. This feature is critical for long-term use in field environmental monitoring where power supply is limited. Unlike high-temperature metal-oxide semiconductor (MOX) sensors, this design reduces energy consumption, enhances mechanical flexibility, and avoids thermal damage to organic matrices (e.g., PANI). These improvements enable its use in wearable and disposable devices while maintaining compatibility with temperature-sensitive organic materials. The sensor demonstrates significant potential for applications in environmental monitoring and wearable technology, offering a sustainable solution for energy-constrained scenarios.

## Figures and Tables

**Table 1:** PANI-based gas-sensitive materials for H_2_S detection.

Type	Materials	OT (°C)	Gas (ppm)	LOD (ppm)	Response	T_res_ (s)	T_rec_ (s)	Ref.
metal	PANI/AuNPs	200	1	-	48%	-	-	[21]
	PANI/Ag	RT	25	-	73.35%	0.82	0.81	[22]
metal oxides	PANI/ZnO	RT	50	0.1	40.5%	63	12	[23]
	PANI/CuO	RT	25	-	188%	>300	-	[24]
	PANI/WO_3_/CuCl_2_	RT	1.156	0.3	93.62%	67.9	250	[25]
	PANI/WO_3_/CuO	RT	1	0.1	31.3%	353	4958	This work
carbon materials	PANI/SnO_2_/rGO	RT	5	0.05	91.11%	80	88	[26]
	PANI/CA	RT	50	1	24.64 (R_a_/R_g_)	1	1065	[27]

## Data Availability

All relevant data are found within the paper.
